# Purification tags markedly affect self‐aggregation of CPEB3

**DOI:** 10.1002/1873-3468.70090

**Published:** 2025-06-17

**Authors:** Harunobu Saito, Yujin Lee, Motoharu Ueno, Naotaka Sekiyama, Masatomo So, Ayako Furukawa, Kenji Sugase

**Affiliations:** ^1^ Division of Applied Life Sciences, Graduate School of Agriculture Kyoto University Japan; ^2^ Department of Biophysics, Graduate School of Science Kyoto University Japan

**Keywords:** amyloid fibril, CPEB3, His tag, IDR, LLPS, protein aggregation

## Abstract

Since protein aggregation—including liquid–liquid phase separation (LLPS) and amyloid fibril formation—plays a critical role in both diseases and biological functions, understanding the mechanisms underlying protein aggregation is essential. Recombinant proteins are commonly used *in vitro* to investigate protein aggregation processes. However, if the purification tags remain uncleaved, they may affect the results and hinder accurate interpretation. Our findings demonstrate that the His_6_‐GFP and His_12_ tags significantly affect liquid droplet and amyloid fibril formation in the intrinsically disordered region (IDR) of mouse cytoplasmic polyadenylation element‐binding protein 3 (CPEB3) and its fragments. This study shows that the purification tags significantly affect aggregation assays, making it essential to account for their influence to accurately interpret protein aggregation.

## Graphical abstract

Although recombinant proteins are used to study protein aggregation *in vitro*, uncleaved tags can interfere with accurate interpretation. Our findings demonstrate that His₆‐GFP and His₁₂ tags significantly affect liquid droplet and amyloid fibril formation in the intrinsically disordered region (IDR) of mouse cytoplasmic polyadenylation element‐binding protein 3 (CPEB3) and its fragments. Our results indicate that tag effects must be considered for accurate interpretation of protein aggregation.

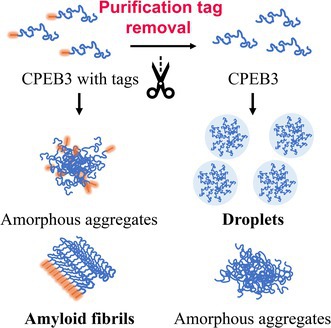

## Abbreviations

**CD**, circular dichroism

**CPEB3**, cytoplasmic polyadenylation element‐binding protein 3

**DIC**, differential interference contrast

**DTT**, dithiothreitol

**Gu‐HCl**, guanidine hydrochloride

**IDR**, intrinsically disordered region

**IMAC**, immobilized metal affinity chromatography

**IPTG**, isopropyl β‐d‐1‐thiogalactopyranoside

**LLPS**, liquid–liquid phase separation

**TEM**, transmission electron microscopy

**ThT**, thioflavin T

**WT**, wild‐type

With the growing interest in phase separation biology, studies of protein aggregates, including liquid droplets and amyloid fibrils, have received increasing attention in recent years [1, 2, 3]. Liquid droplets play a crucial role in diverse biological processes, such as transcription, chromatin organization, autophagy, and signal transduction [4, 5, 6, 7], by promoting compartmentalization and creating specialized reaction environments. Furthermore, reversible droplet formation can transition into solid, irreversible amyloid fibrils that are implicated in neurodegenerative diseases [8, 9, 10, 11]. Notably, recent studies have indicated that certain amyloid fibrils are not solely pathogenic but also play essential physiological roles [12, 13, 14]. Recognizing that protein aggregation plays a critical role in both pathological and physiological processes, elucidating the mechanisms that underlie its formation remains crucial. To achieve this, recombinant protein aggregation assays have been extensively utilized as a primary approach.

In many studies, purification tags are retained during analysis [15, 16, 17, 18]. The His tag is one of the most commonly used tags due to its low molecular weight and ease of purification through immobilized metal affinity chromatography (IMAC), such as Ni‐NTA [19]. However, the His tag consists of a series of histidine residues, which can alter the protein's charge distribution and thermal stability [20]. This is particularly significant for intrinsically disordered regions (IDRs), where aggregation is driven by multivalent interactions, including electrostatic, cation–π, π–π, and dipole interactions [21, 22, 23, 24]. As a result, the presence of a His tag can have a pronounced impact on protein aggregation. Moreover, as the p*K*a of histidine is near the pH range typically used in experimental conditions mimicking physiological pH, its charge state is likely to affect aggregation behavior.

In fact, previous studies have reported ambiguous interpretations of protein aggregation behavior that may be attributed to the influence of the His tag on proteins with IDRs [15, 16, 17, 18]. This uncertainty highlights the need to reassess aggregation phenomena in specific proteins of biological interest. In this context, cytoplasmic polyadenylation element‐binding protein 3 (CPEB3) emerges as a compelling model. CPEB3 is an RNA‐binding protein that is essential for the formation of long‐term memory [25]. In neurons, CPEB3 regulates the localized synaptic translation of AMPA‐type glutamate receptor subunits and actin, thereby supporting critical physiological processes such as synaptic plasticity and the maintenance of long‐term memory [25, 26]. The translational regulation of CPEB3 is believed to be modulated by its aggregation state, with previous studies demonstrating its capacity to form both droplets and amyloid fibrils *in vitro* [14, 15, 17, 18].

In this study, we demonstrate that the aggregation behavior of the CPEB3 IDR and the two isolated prion‐like fragments contained within it is significantly affected by the presence of either the His_6_‐GFP tag or the His_12_ tag. To enable the efficient removal of purification tags from aggregation‐prone IDRs, we developed a tag cleavage protocol under mildly denaturing conditions. Given that many proteins contain aggregation‐prone IDRs, the influence of purification tags is not limited to CPEB3, suggesting a potential general issue in protein aggregation studies.

## Materials and methods

### Protein expression and purification

The mouse *CPEB3* [1–459] gene was subcloned into the pET His_6_‐GFP‐TEV LIC vector (Addgene, Watertown, MA, USA). The GFP variant used in this construct is the superfolder GFP [27], which was engineered through multiple mutations to enhance brightness, improve solubility, and prevent dimerization. For protease cleavage analysis, an additional HRV3C cleavage site was inserted between the His_6_‐GFP tag and CPEB3 [1–459]. Consequently, the resulting construct was His_6_‐GFP‐(TEV cleavage site)‐(HRV3C cleavage site)‐CPEB3 [1–459]. The mouse *CPEB3* [101–200] and *CPEB3* [294–410] genes were subcloned into the pET‐28b vector using In‐Fusion cloning. Additionally, a His_12_ tag and a TEV cleavage site were added at the N terminus. The human *α‐synuclein* gene was subcloned into the pET‐15b vector. The human *α‐synuclein* with an N‐terminal His_6_ tag was subcloned into the pET‐ST‐2 vector.

Detailed procedures for protein expression and purification are provided in the Supporting Information; briefly, the CPEB3 and α‐synuclein constructs were expressed in *Escherichia coli* BL21(DE3) cultured in LB medium. α‐Synuclein was purified as previously described [28]. The CPEB3 proteins were expressed as inclusion bodies. They were solubilized in 50 mm Tris/HCl buffer (pH 8.0) containing either 8 M urea or 6 M guanidine‐HCl (Gu‐HCl) and purified using a HisTrap HP column (Cytiva, Wilmington, DE, USA). Portions of the purified proteins before tag cleavage were retained as controls. The remaining proteins were then dialyzed against appropriate buffers. His_6_‐TEV protease was then added, and the mixture was incubated overnight at 25 °C. Tag cleavage efficiency was evaluated as described below in the protease activity assay. The cleaved tags and protease were removed by additional column purification. Protein purity was assessed by SDS/PAGE or MALDI‐TOF MS. Protein concentrations were determined by measuring the absorbance at 280 nm or the BCA Protein Assay Kit (Takara, Kusatsu, Shiga, Japan).

### Protease activity assay

To evaluate protease activity in the presence of urea or Gu‐HCl, purified His_6_‐GFP‐CPEB3 [1–459] was diluted to 0.5 mg·mL^−1^ in 20 mm Tris/HCl (pH 8.0), 0.5 mm EDTA, and 1 mm DTT, and supplemented with 1–4 M urea or 0.5–2 M Gu‐HCl. His_6_‐TEV protease or GST‐HRV3C protease was added at a protease‐to‐substrate 1 : 50 (w/w) ratio, and the reaction was incubated overnight at 25 °C. The TEV protease used in this study contains mutations that enhance its stability and specificity [29, 30, 31, 32, 33, 34]. Proteolytic cleavage efficiency was evaluated by SDS/PAGE.

### Fluorescence and differential interference contrast microscopy

To induce protein aggregation, a 3 mm protein sample, originally in 20 mm phosphate buffer (pH 7.4) containing 3 M Gu‐HCl, was diluted 60‐fold into 10 mm phosphate buffer (pH 7.4) containing 50 mm NaCl, resulting in a final protein concentration of 50 μm. For CPEB3 [1–459] (with or without the His_6_‐GFP tag), the dilution buffer additionally included 1 mm DTT. For CPEB3 [294–410] (with or without the His_12_ tag), the dilution buffer included 10% PEG‐8000 (Sigma‐Aldrich, St. Louis, MO, USA). A 4 μL aliquot of the sample was placed into a sample chamber assembled using double‐sided tape with prepunched holes, mounted on a glass slide, and sealed with a coverslip. Bright‐field and fluorescence imaging were performed using an Axio Observer confocal microscope (Zeiss, Jena, Thuringia, Germany) equipped with a Plan‐Apochromat 63×/1.4 oil differential interference contrast (DIC) objective. For fluorescence microscopy, GFP fluorescence was excited at 488 nm

### Turbidity assay

A solution of 2.5 mm CPEB3 [294–410] (with or without the His_12_ tag), prepared in 20 mm phosphate buffer (pH 7.4) containing 3 M Gu‐HCl, was diluted 50‐fold into 10 mm phosphate buffer (pH 7.4) containing 50 mm NaCl and 10% PEG‐8000, resulting in a final protein concentration of 50 μm. The dilution buffer was preheated to 95 °C prior to sample mixing. The diluted samples were subsequently incubated at 95 °C for 5 min. Turbidity was monitored at 600 nm using a V‐750 UV–Vis spectrometer (JASCO, Hachioji, Tokyo, Japan) equipped with a PAC‐743 water‐cooled Peltier cell changer. The temperature was then decreased from 95 to 4 °C at a rate of 1 °C·min^−1^.

### Thioflavin T fluorescence assay

A solution of 3 mm CPEB3 [126–169] (with or without the His_12_ tag) and 3 mm α‐synuclein (with or without the His_6_ tag), prepared in 50 mm MES buffer (pH 5.5) containing 3 M Gu‐HCl, was diluted 60‐fold into 50 mm MES buffer (pH 5.5), resulting in a final protein concentration of 50 μm. The sample was continuously vortexed at 37 °C for 96 h. About 5 μL of the sample was added to 1 mL of 50 mm MES buffer (pH 5.5) containing 25 μm ThT. Thioflavin T (ThT) fluorescence was recorded from 440 to 550 nm using an FP‐6500 fluorometer (JASCO) with excitation at 445 nm.

### Fluorescence measurement of phenylalanine

The CPEB3 [126–169] (with or without the His_12_ tag) samples, prepared as described in the ThT fluorescence assay, were diluted 10‐fold into 50 mm MES buffer (pH 5.5). Phenylalanine fluorescence was recorded from 250 to 450 nm using an FP‐6500 fluorometer (JASCO) with excitation at 225 nm.

### Circular dichroism measurement

The CPEB3 [126–169] (with or without the His_12_ tag) samples were prepared as described in the ThT fluorescence assay. Circular dichroism (CD) spectra were recorded from 199 to 250 nm using a J‐750 circular dichroism spectrometer (JASCO) with a 0.5‐mm path length quartz cell.

### Transmission electron microscopy

The aggregates formed in the ThT fluorescence assay were collected by centrifugation and diluted fivefold with Milli‐Q water. Immediately after dilution, the sample was spotted onto a hydrophilized carbon grid and stained with EM‐Stainer (Nisshin EM, Shinjuku, Tokyo, Japan). Following two washes with Milli‐Q water, the grid was examined using a JEM‐1400 (JEOL, Akishima, Tokyo, Japan) transmission electron microscope.

## Results and discussion

### Preparation of untagged CPEB3 from inclusion bodies

**Fig. 1:**
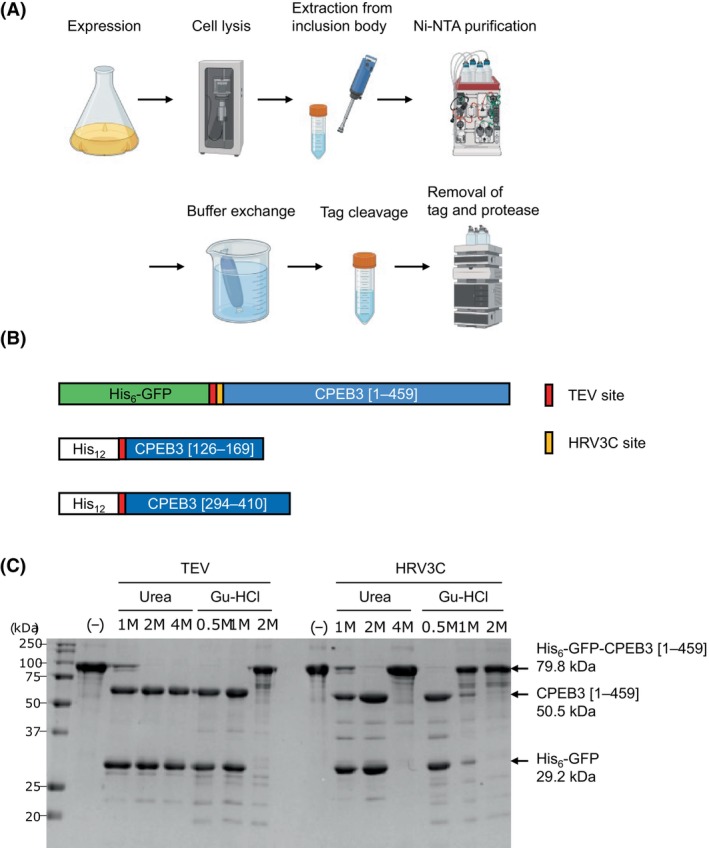
Preparation of untagged CPEB3. (A) Schematic representation of the purification and tag removal processes for preparing untagged CPEB3. (B) The CPEB3 constructs used in this study. (C) Protease‐mediated tag cleavage activity under denaturing conditions. TEV and HRV3C proteases were assessed for cleavage activity at various concentrations of urea and Gu‐HCl using the His_6_‐GFP‐CPEB3 [1–459] construct, which contains both TEV and HRV3C cleavage sites between the His_6_‐GFP tag and CPEB3 [1–459]. Representative result is shown.

We developed a protocol to purify untagged CPEB3 from inclusion bodies by cleaving off the purification tags under denaturing conditions to evaluate their impact on aggregation (Fig. 1A). We constructed vectors encoding His_6_‐GFP‐CPEB3 [1–459], His_12_‐CPEB3 [126–169], and His_12_‐CPEB3 [294–410] (Fig. 1B) for efficient purification using IMAC resin [35, 36]. Here, CPEB3 [1–459] comprises the entire IDR of CPEB3, while CPEB3 [126–169] and CPEB3 [294–410] correspond to two prion‐like domains [26]. Specifically, CPEB3 [126–169] is likely to form amyloid fibrils, whereas CPEB3 [294–410] is implicated in droplet formation [17]. Since purification of the CPEB3 from the soluble fraction proved challenging due to the high aggregation propensity, we purified CPEB3 from the insoluble fraction. After bacterial expression, the inclusion bodies were solubilized with either 8 M urea or 6 M Gu‐HCl and then purified by Ni‐NTA affinity chromatography.

To optimize the tag cleavage conditions, we assessed the activity of TEV and HRV3C proteases using His_6_‐GFP‐CPEB3 [1–459], which contains both TEV and HRV3C cleavage sites. Both proteases retained activity in the presence of low concentrations of denaturants (Fig. 1C), consistent with previous reports [37, 38]. TEV protease remained active up to 4 M urea and 1 M Gu‐HCl, while HRV3C protease showed activity at 2 M urea and 0.5 M Gu‐HCl. Under 1 M urea conditions, however, the sample remained uncleaved after overnight incubation, likely due to aggregation of the protein. Furthermore, HRV3C protease caused more nonspecific cleavage than TEV protease. This may be due to the lack of a defined structure in CPEB3, which makes it more susceptible to nonspecific cleavage. Based on these results, we selected TEV protease for subsequent experiments and performed tag cleavage in the TEV reaction buffer containing 2 M urea or 1 M Gu‐HCl. The cleaved tag and TEV protease were then removed via additional column purification.

Note that omitting EDTA from the TEV reaction buffer resulted in reduced cleavage efficiency and the formation of visible aggregates (data not shown). This suggests that Ni^2+^ ions leaking from the Ni‐NTA column promoted the aggregation of CPEB3. Such aggregation may be initiated by coordination between Ni^2+^ and the His_6_ tag, a phenomenon previously reported for other His‐tagged proteins [39, 40]. In addition, previous studies have demonstrated that various IDRs in proteins, such as those in α‐synuclein, tau, and TIA‐1 [41, 42, 43, 44], interact with metal ions via their polar and negatively charged amino acid‐rich sequences. These studies indicated that electrostatic interactions between the metal ions and the proteins caused aggregation. Similarly, the polar and negatively charged residues in CPEB3 may coordinate with metal ions like Ni^2+^, especially in the presence of the His_6_ tag, further promoting aggregation. Therefore, it is crucial to remove Ni^2+^ ions using EDTA.

### His_6_‐GFP tag‐induced alteration of CPEB3 [1–459] aggregation morphology

**Fig. 2:**
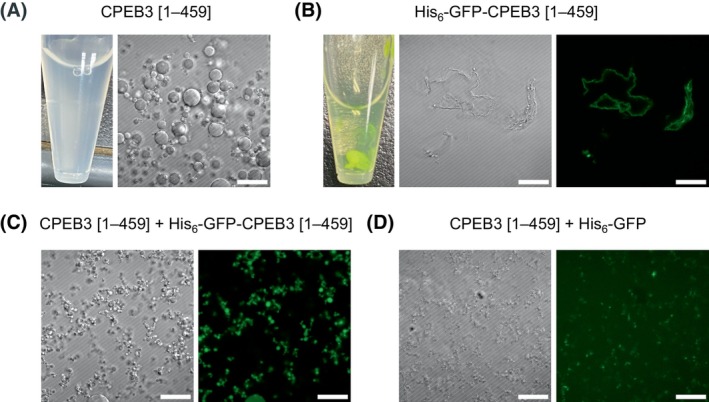
Effect of the His_6_‐GFP tag on the aggregation morphology of CPEB3 [1–459]. (A) CPEB3 [1–459] solution and a DIC image of its aggregates. (B) His_6_‐GFP‐CPEB3 [1–459] solution and DIC and fluorescence images of its aggregates. (C) DIC and fluorescence images of a mixture of CPEB3 [1–459] and His_6_‐GFP‐CPEB3 [1–459]. (D) DIC and fluorescence images of a mixture of CPEB3 [1–459] and His_6_‐GFP. The scale bar is 20 μm. Images shown are representative examples.

To evaluate the impact of the His_6_‐GFP tag on the aggregation morphology of CPEB3 [1–459], we analyzed the aggregation properties of CPEB3 [1–459], which represents the entire IDR of CPEB3. Aggregation was induced by rapidly diluting the denatured His_6_‐GFP‐CPEB3 [1–459] (dissolved in 3 M Gu‐HCl) into a native buffer containing 10 mm phosphate (pH 7.4) and 50 mm NaCl. As a result, the CPEB3 [1–459] solution became uniformly cloudy, and droplets were observed by DIC microscopy (Fig. 2A). In contrast, when His_6_‐GFP‐CPEB3 [1–459] was subjected to the same process, it did not form droplets but instead produced amorphous aggregates (Fig. 2B). The altered aggregation behavior of His_6_‐GFP‐CPEB3 [1–459] likely resulted from interactions between the His_6_‐GFP tag and CPEB3 [1–459], rather than from the inherent solubility of the His_6_‐GFP tag [27] or GFP misfolding. Note that His_6_‐GFP‐CPEB3 [1–459] retained its GFP fluorescence both in 3 M Gu‐HCl and in the native buffer, indicating that the GFP remained structurally intact.

When His_6_‐GFP‐CPEB3 [1–459] was added to CPEB3 [1–459] at a 5% molar ratio, the droplets decreased in size and became more tightly aggregated (Fig. 2C). This suggests that intermolecular interactions between His_6_‐GFP‐CPEB3 [1–459] and CPEB3 [1–459] promote the formation of rigid aggregates, with contributions from both the His_6_‐GFP tag and the CPEB3 [1–459] region. Notably, the addition of an equimolar amount of the isolated His_6_‐GFP tag to CPEB3 [1–459] significantly altered aggregate morphology, converting droplets into amorphous structures (Fig. 2D). Since the amorphous aggregates exhibited fluorescence, the His_6_‐GFP tag was incorporated into the aggregates of CPEB3 [1–459] and altered their aggregation properties.

### His_12_ tag‐induced alteration of amyloid fibril structure of CPEB3 [126–169]

**Fig. 3:**
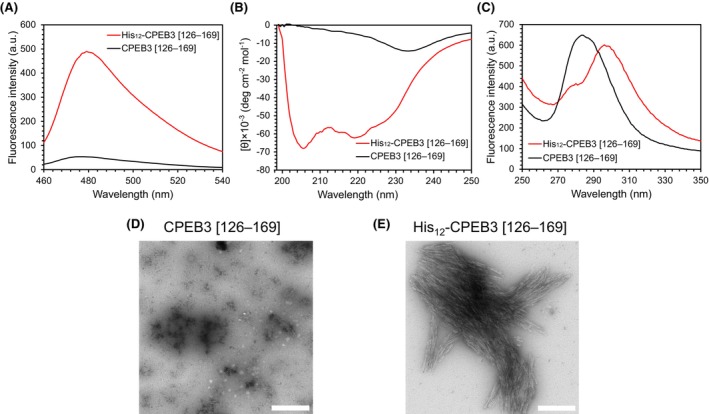
Effect of the His_12_ tag on amyloid fibril structure of CPEB3 [126–169]. (A) ThT fluorescence assay of CPEB3 [126–169] and His_12_‐CPEB3 [126–169]. (B) The CD spectra of CPEB3 [126–169] and His_12_‐CPEB3 [126–169]. (C) The phenylalanine fluorescence spectra of CPEB3 [126–169] and His_12_‐CPEB3 [126–169]. In His_12_‐CPEB3 [126–169], tyrosine fluorescence from the TEV cleavage site was also observed. The presented result is representative of repeated experiments. TEM images of aggregates of (D) CPEB3 [126–169] and (E) His_12_‐CPEB3 [126–169]. The scale bar is 400 nm. Images shown are representative examples.

Since CPEB3 [126–169] is likely to form amyloid fibrils [17], we evaluated the effect of the His_12_ tag on fibril formation by comparing the ThT fluorescence of His_12_‐CPEB3 [126–169] with that of the untagged sample. His_12_‐CPEB3 [126–169] displayed enhanced ThT fluorescence relative to the untagged sample (Fig. 3A), suggesting that His_12_‐CPEB3 [126–169] forms more stable fibrils than does the untagged sample. To confirm that the ThT fluorescence originated from amyloid fibrils, we measured the CD spectra of CPEB3 [126–169] with and without the His_12_ tag (Fig. 3B). Remarkably, His_12_‐CPEB3 [126–169] exhibited pronounced negative peaks at 206 and 220 nm, indicative of an α‐helical structure characteristics of cross‐α amyloid fibrils, and showed no detectable β‐sheet formation, in contrast to typical cross‐β amyloid fibrils. On the other hand, the untagged CPEB3 [126–169] exhibited a weaker peak at 233 nm, suggesting the presence of a minor antiparallel β‐sheet structure in the randomly formed aggregates.

Furthermore, we measured the phenylalanine fluorescence of the aggregates, as CPEB3 [126–169] contains six phenylalanine residues. The fluorescence spectrum of untagged CPEB3 [126–169] showed the typical pattern of free phenylalanine in solution, whereas His_12_‐CPEB3 [126–169] exhibited a red shift (Fig. 3C), suggesting a conformational difference that alters the local environment of the phenylalanine residues. We also examined the aggregate morphology of the samples using transmission electron microscopy (TEM; JEOL) and revealed the presence of amyloid fibrils in His_12_‐CPEB3 [126–169] (Fig. 3D), whereas most aggregates of untagged CPEB3 [126–169] appeared as amorphous structures or oligomers (Fig. 3E).

These results suggest that the His_12_ tag promotes the formation of cross‐α amyloid fibrils in CPEB3 [126–169]. To date, only a limited number of amyloid fibrils with a cross‐α structure have been reported [45, 46, 47]. Notably, all amyloid fibrils formed by homologs of CPEB3 lacking the purification tags have exhibited cross‐β‐sheet structures [14, 15]. Therefore, the His_12_ tag significantly influences amyloid fibril structures.

Given that CPEB3 [126–169] contains six phenylalanine residues, four histidine residues, and seven glutamine residues, cation‐π and electrostatic interactions may play important roles in the aggregation process. The aforementioned experiments were conducted at pH 5.5, a value below the p*K*a of histidine, indicating that the His residues are protonated and that the His_12_ tag could have modulated the intermolecular interactions via charge‐mediated effects. Taken together, these findings suggest that the His_12_ tag alters CPEB3 [126–169] fibril structure at pH 5.5 via charge‐mediated effects, favoring cross‐α over cross‐β forms.

### His_12_ tag‐induced alteration of the aggregation state of CPEB3 [294–410] from droplets to amorphous aggregates

**Fig. 4:**
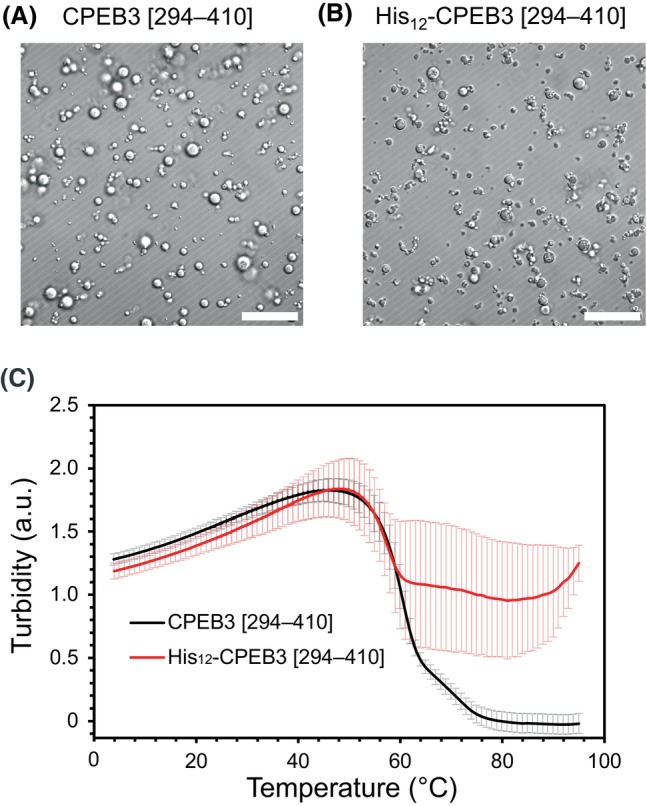
Effect of His_12_ tag on LLPS of CPEB3 [294–410]. The DIC images of (A) CPEB3 [294–410] and (B) His_12_‐CPEB3 [294–410]. The scale bar is 20 μm. Images shown are representative examples. (C) Turbidity assay of CPEB3 [294–410] and His_12_‐CPEB3 [294–410]. *N* = 3. Error bars represent the standard deviation.

Since CPEB3 [294–410] is implicated in droplet formation [17], we evaluated the effect of the His_12_ tag on the aggregate morphology by comparing the DIC image of His_12_‐CPEB3 [294–410] with that of the untagged sample. Consistent with our previous observations for CPEB3 [1–459] (in the absence of PEG‐8000), the His_12_ tag likewise promoted droplet formation for CPEB3 [294–410] in the presence of 10% PEG‐8000 (Fig. 4A). In contrast, His_12_‐CPEB3 [294–410] formed droplets that retained droplet‐like features while aggregating more extensively, resulting in a morphology reminiscent of amorphous aggregates (Fig. 4B).

We also conducted turbidity assays to determine whether the droplets were formed via LLPS, a process that is often reversible with temperature changes. In this assay, measurements began at 95 °C and the temperature was gradually decreased. Under these conditions, CPEB3 [294–410] exhibited low turbidity at high temperatures, followed by a rapid increase in turbidity upon cooling (Fig. 4C). In contrast, His_12_‐CPEB3 [294–410] showed high turbidity at 95 °C and displayed only a modest increase in turbidity as the temperature decreased. Once the turbidity of His_12_‐CPEB3 [294–410] began to rise sharply, its subsequent turbidity profile closely matched that of untagged CPEB3 [294–410].

The DIC images and turbidity results suggest that the aggregates formed by the CPEB3 [294–410] region in His_12_‐CPEB3 [294–410] are similar to those formed by CPEB3 [294–410] alone, while the His_12_ tag appears to promote additional interactions. Given that the CPEB3 [294–410] region also contains six histidine residues, it is likely that the His_12_ tag alters the intermolecular interactions essential for LLPS by excessively promoting histidine‐mediated contacts. Taken together, these findings suggest that the His_12_ tag disrupts typical LLPS, leading to the formation of amorphous aggregates.

### His_6_ tag‐suppressed formation of α‐synuclein amyloid fibrils

**Fig. 5:**
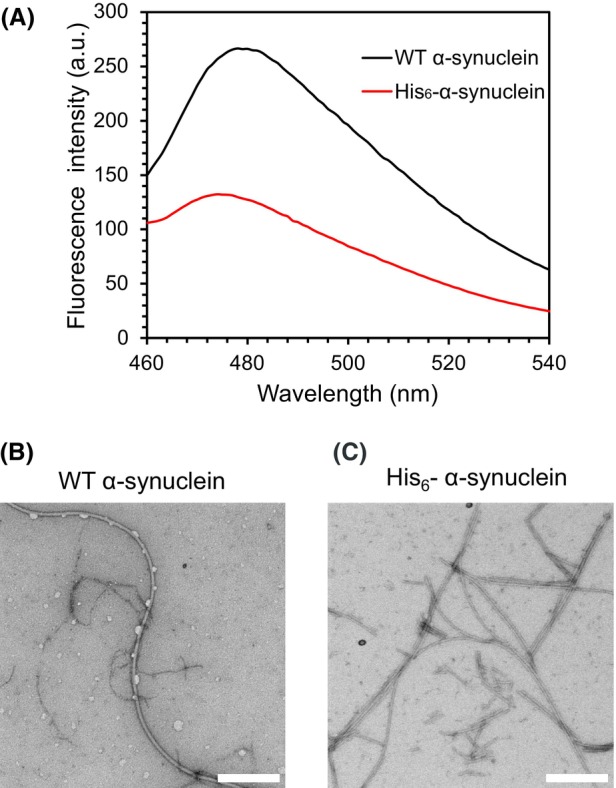
Effect of His_6_ tag on amyloid fibril formation of α‐synuclein. (A) ThT fluorescence assay of WT α‐synuclein and His_6_‐α‐synuclein. The presented result is representative of repeated experiments. TEM images of aggregates of (B) WT α‐synuclein and (C) His_6_‐α‐synuclein. The scale bar is 400 nm. Images shown are representative examples.

To examine whether the purification tag similarly affects the aggregation of other proteins, we evaluated the effect of the His_6_ tag on α‐synuclein fibril formation using ThT fluorescence measurements. His_6_‐α‐synuclein exhibited lower ThT fluorescence intensity than WT α‐synuclein (Fig. 5A). Furthermore, TEM imaging confirmed that His_6_‐α‐synuclein formed amyloid fibrils similar to those formed by WT α‐synuclein (Fig. 5B,C). These findings suggest that the His_6_ tag partially suppresses the formation of α‐synuclein amyloid fibrils.

## Conclusion

In this study, we explored the impact of the purification tag on the self‐aggregation of CPEB3. Both the His_6_‐GFP and His_12_ tags appear to enhance the aggregation properties of CPEB3, which is consistent with previous studies reporting increased β‐sheet formation, oligomerization, and decreased thermal stability [20, 48, 49]. Consequently, the CPEB3 droplets became amorphous, and its oligomers transitioned into amyloid fibrils. Furthermore, the His_6_ tag counteracted the solubility‐enhancing effects of the fused GFP tag, while the His_12_ tag significantly altered the structure of the resulting amyloid fibrils. Given that the IDR of CPEB3 contains as many as 14 histidine residues, it is plausible that the His tag modulates intra‐ and intermolecular interactions through these histidine residues. Indeed, a histidine cluster has been shown to be crucial for the formation of both amyloid fibrils and droplets in Orb2 (the *Drosophila* ortholog) and CPEB4 [14, 50]. However, the detailed mechanism of the tag effect remains to be elucidated.

*In vitro* characterization of aggregates is essential for understanding their formation *in vivo* and their potential contributions to function and disease. However, our study highlights the necessity of eliminating the influence of the purification tag during the experimental characterization of aggregates. The His tag, widely employed in recombinant protein production due to its ease of purification via Ni‐NTA chromatography [19], is typically assumed to have minimal effect on protein structure and function due to its small molecular weight. However, caution is warranted when interpreting experiments on the aggregation properties and structural characteristics of CPEB3 conducted without prior removal of the His tag [15, 16, 17, 18]. Furthermore, the effect of the purification tag may not be negligible in studies with other proteins related to LLPS or amyloid fibril formation, such as ataxin‐2, hnRNPA1, TDP‐43, and TIA‐1 [51, 52, 53, 54, 55]. It may similarly influence the aggregation of other proteins such as α‐synuclein, amyloid‐β, huntingtin, and prion protein [11, 56, 57, 58]. Indeed, we showed that the His_6_ tag partially suppressed α‐synuclein fibril formation, whereas a previous study reported that it promoted amyloid‐β fibril formation [59]. These effects must be considered when discussing *in vivo* function and diverse disease phenotypes related to amyloid polymorphism and LLPS.

Additionally, the purification scheme established in this study, which employs TEV protease that retains strong activity even under denaturing conditions, may prove valuable for future studies involving aggregation‐prone IDRs such as ataxin‐2, hnRNPA1, TDP‐43, and TIA‐1. The tag cleavage during the purification process also reduces aggregation, potentially simplifying protein purification. External factors such as nucleic acids, metal ions, ATP, and pH fluctuations are known to contribute to aggregation [10, 39, 40, 50, 60], making it preferable to remove tags when studying the association between these factors and aggregation *in vitro*. In conclusion, our study provides compelling evidence of the significant impact that the purification tag can have on protein aggregation, emphasizing the need for careful consideration of this factor in future studies.

## Conflict of interest

The authors declare no conflict of interest.

## Author contributions

HS, YL, and MU conducted the experiment. HS wrote the main manuscript text and prepared all figures. NS, MS, and AF contributed to project supervision, while KS oversaw the project.

## Peer review

The peer review history for this article is available at https://www.webofscience.com/api/gateway/wos/peer‐review/10.1002/1873‐3468.70090.

## Supporting information


**Supporting Information**. Detailed Materials and Methods.

## Data Availability

The data that support the findings of this study are available from the corresponding author upon reasonable request.
