# Supplementary material for: Purification tags markedly affect self‐aggregation of CPEB3
Source: FEBS Lett. 2025 Jun 17;599(19):2779–89. doi: 10.1002/1873-3468.70090 (PMC12519062; doi:10.1002/1873-3468.70090)
Supplement: Supplementary file 1 — Supporting Information. Detailed Materials and Methods. [file FEB2-599-2779-s001.docx]

**Purification tags markedly affect self-aggregation of CPEB3**

Harunobu Saito^1^, Yujin Lee^1^, Motoharu Ueno^2^, Naotaka Sekiyama^2^, Masatomo So^1^, Ayako Furukawa^1^, Kenji Sugase^1^

1: Division of Applied Life Sciences, Graduate School of Agriculture, Kyoto University

2: Department of Biophysics, Graduate School of Science, Kyoto University

**Supporting Information**

Detailed Materials and Methods

**Detailed Materials and methods**

**Protein expression and purification**

**CPEB3 constructs**

*Escherichia coli* strain BL21(DE3) was transformed with the respective expression vectors, and the cells were cultured in LB medium supplemented with 25 mg/L kanamycin (Wako) at 37 °C. Protein expression was induced with 1 mM isopropyl β-D-1-thiogalactopyranoside (IPTG; Nacalai Tesque) at an OD_600_ of 0.8. The cultures were incubated at 37 °C for 3 hours for His_6_-GFP-CPEB3 [1–459] and His_12_-CPEB3 [294–410], while His_12_-CPEB3 [126–169] was expressed at 20 °C overnight. After incubation, the cells were harvested by centrifugation, resuspended in lysis buffer (50 mM Tris-HCl (pH 8.0), 50 mM NaCl, 5 mM β-mercaptoethanol, 0.1 mM PMSF, and 0.1% Triton X-100), and lysed by sonication on ice using a Q500 (Q-Sonica). The lysate was subsequently centrifuged, and the inclusion body pellet was washed with wash buffer (50 mM Tris-HCl (pH 8.0), 50 mM NaCl, 2 M urea, and 0.5% Triton X-100) and then centrifuged.

For the purification of His_6_-GFP-CPEB3 [1–459], the inclusion bodies were resuspended in denaturing buffer (50 mM Tris-HCl (pH 8.0), 6 M guanidine-HCl (Gu-HCl), 5 mM imidazole, and 1 mM dithiothreitol (DTT)). The sample solution was centrifuged, and the supernatant was filtered through a 0.45-µm filter and then loaded onto a HisTrap HP column (Cytiva). The column was washed with five column volumes of the denaturing buffer, and the bound protein was eluted with 200 mM imidazole. A portion of the eluted His_6_-GFP-tagged protein was retained without tag cleavage. The buffer was exchanged with 20 mM phosphate buffer (pH 7.4) containing 3 M Gu-HCl and 1 mM DTT, concentrated to 3 mM using an Amicon Ultra centrifugal filter (Merck), and stored at –80 °C. The remaining eluted protein was subjected to cleavage of the His_6_-GFP tag under various conditions, as described in the protease activity assay below. After cleavage, 8 M urea was added, and the sample was loaded onto a HiTrap Q HP column (Cytiva). The column was washed with two column volumes of 20 mM Tris-HCl buffer (pH 8.0) containing 8 M urea, 50 mM NaCl, and 1 mM DTT, and the protein was eluted using a 50–500 mM NaCl gradient. The sample was further purified using a HiLoad 16/600 Superdex 200 pg column (Cytiva) and eluted with 20 mM phosphate buffer (pH 7.4) containing 3 M Gu-HCl and 1 mM DTT. The purified protein was concentrated to 3 mM using an Amicon Ultra centrifugal filter (Merck) and stored at –80 °C. To prevent aggregation, samples were thawed immediately before experiments, and repeated freeze-thaw cycles were avoided.

For His_12_-CPEB3 [126–169] and His_12_-CPEB3 [294–410], the inclusion bodies were resuspended in denaturing buffer (50 mM Tris-HCl (pH 8.0) and 5 mM imidazole) containing either 6 M Gu-HCl (for His_12_-CPEB3 [294–410]) or 8 M urea (for His_12_-CPEB3 [126–169]). The sample solution was centrifuged, and the supernatant was filtered through a 0.45-µm filter and then loaded onto a HisTrap HP column (Cytiva). The column was washed with five column volumes of the denaturing buffer, and the bound protein was eluted with a 5–500 mM imidazole gradient. A portion of the eluted His_12_-tagged proteins was retained without tag cleavage. The buffer was exchanged with 10 mM HCl, and the sample was lyophilized. The remaining eluted proteins were dialyzed against TEV reaction buffer (20 mM Tris-HCl (pH 8.0), 2 M urea, 0.5 mM EDTA, and 1 mM DTT for His_12_-CPEB3 [126–169]; 20 mM MES (pH 6.0), 1 M Gu-HCl, 0.5 mM EDTA, and 1 mM DTT for His_12_-CPEB3 [294–410]). Insoluble aggregates were then removed by centrifugation. His_6_-TEV protease was added, and the mixture was incubated overnight at 25 °C. For CPEB3 [126–169], the cleaved His_12_ tag and protease were removed using C18 reverse-phase HPLC, and the purified CPEB3 [126–169] was lyophilized. For CPEB3 [294–410], the cleaved His_12_ tag and protease were removed using a HisTrap HP column (Cytiva), and the protein was eluted with a 5–500 mM imidazole gradient. The purified CPEB3 [294–410] was then dialyzed against 10 mM HCl and lyophilized. The resulting protein powder was dissolved in 3 M Gu-HCl solution containing 50 mM MES (pH 5.5) or 20 mM phosphate (pH 7.4) immediately before each experiment. Protein concentrations were determined by measuring the absorbance at 280 nm using a DS-11 spectrophotometer (DeNovix). The extinction coefficients (ε_280_) were 41,940 for CPEB3 [1–459], 62,340 for His_6_-GFP-CPEB3 [1–459], 13,980 for CPEB3 [294–410], and 15,470 for His_12_-CPEB3 [294–410]. For CPEB3 [126–169] and His_12_-CPEB3 [126–169], protein concentrations were determined using the BCA Protein Assay Kit (Takara) due to their lack of absorbance at 280 nm.

**α-synuclein constructs**

*Escherichia coli* strain BL21(DE3) was transformed with the respective expression vectors, and the cells were cultured in LB medium supplemented with 50 mg/L ampicillin (Wako) at 37 °C. Protein expression was induced with 1 mM IPTG at an OD_600_ of 0.6. The cultures were incubated at 25 °C for 16 hours. After incubation, the cells were harvested by centrifugation. Purification of α-synuclein constructs was carried out as previously described [1]. Protein purity was assessed by SDS-PAGE. Protein concentrations were determined by measuring the absorbance at 280 nm using a DS-11 spectrophotometer (DeNovix). The extinction coefficient (ε_280_) was 5,960 for both WT α-synuclein and His_6_-α-synuclein.

**Reference**

1 Yagi H, Kusaka E, Hongo K, Mizobata T and Kawata Y (2005) Amyloid fibril formation of alpha-synuclein is accelerated by preformed amyloid seeds of other proteins: implications for the mechanism of transmissible conformational diseases. *J Biol Chem* **280**, 38609–38616.
